# Neuronal Nitric Oxide Synthase-Rescue of Dystrophin/Utrophin Double Knockout Mice does not Require nNOS Localization to the Cell Membrane

**DOI:** 10.1371/journal.pone.0025071

**Published:** 2011-10-07

**Authors:** Michelle Wehling-Henricks, James G. Tidball

**Affiliations:** 1 Department of Integrative Biology and Physiology, University of California, Los Angeles, California, United States of America; 2 Molecular, Cellular and Integrative Physiology Program, University of California, Los Angeles, California, United States of America; 3 Department of Pathology and Laboratory Medicine, David Geffen School of Medicine, University of California, Los Angeles, California, United States of America; University of Queensland, Australia

## Abstract

Survival of dystrophin/utrophin double-knockout (dko) mice was increased by muscle-specific expression of a neuronal nitric oxide synthase (nNOS) transgene. Dko mice expressing the transgene (nNOS TG+/dko) experienced delayed onset of mortality and increased life-span. The nNOS TG+/dko mice demonstrated a significant decrease in the concentration of CD163+, M2c macrophages that can express arginase and promote fibrosis. The decrease in M2c macrophages was associated with a significant reduction in fibrosis of heart, diaphragm and hindlimb muscles of nNOS TG+/dko mice. The nNOS transgene had no effect on the concentration of cytolytic, CD68+, M1 macrophages. Accordingly, we did not observe any change in the extent of muscle fiber lysis in the nNOS TG+/dko mice. These findings show that nNOS/NO (nitric oxide)-mediated decreases in M2c macrophages lead to a reduction in the muscle fibrosis that is associated with increased mortality in mice lacking dystrophin and utrophin. Interestingly, the dramatic and beneficial effects of the nNOS transgene were not attributable to localization of nNOS protein at the cell membrane. We did not detect any nNOS protein at the sarcolemma in nNOS TG+/dko muscles. This important observation shows that sarcolemmal localization is not necessary for nNOS to have beneficial effects in dystrophic tissue and the presence of nNOS in the cytosol of dystrophic muscle fibers can ameliorate the pathology and most importantly, significantly increase life-span.

## Introduction

Duchenne muscular dystrophy (DMD) is an inherited, fatal disease of muscle wasting that affects 1 in 3500 newborn males [1]. DMD patients experience progressive muscle atrophy, which results in loss of function, respiratory insufficiencies and cardiac abnormalities [1], [2]. Most patients lose the ability to ambulate by age 12 [2], begin using ventilatory assistance in the late teens [3], and all patients suffer from clinical cardiac involvement by age 18 [4]. There is no cure or well-tolerated treatment for DMD and death typically occurs in the late teens or early twenties due to respiratory or cardiac complications [2], [4]. The genetic basis for DMD is a mutation of the dystrophin gene that prevents expression of dystrophin protein [5]. Dystrophin is normally localized beneath the sarcolemma where it anchors a membrane-spanning protein complex, the dystrophin glycoprotein complex (DGC), which links the cytoskeleton to the extracellular matrix and provides membrane stabilization [6]. Absence of dystrophin results in loss of the DGC proteins and destabilization of the sarcolemma making the muscle cell membrane more susceptible to mechanical damage and subsequent necrosis [7], [8]. The muscle pathology is exacerbated by phenotypically-distinct populations of inflammatory cells that lyse muscle cells and promote pathological fibrosis. The greatest proportion of invading immune cells are classically-activated, M1 macrophages that lyse muscle cells via inducible nitric oxide synthase (iNOS)-mediated mechanisms [9]. Also present in dystrophic muscle are arginase-expressing, M2 macrophages that are associated with repair processes and promote connective tissue deposition to drive the pathological fibrosis that is characteristic of the disease [9], [10]. Ultimately, deficiencies in the regenerative capacity of dystrophin-deficient muscle preclude normal repair of the tissue and the necrotic fibers are eventually replaced with connective tissue that permanently compromises function [11].

Secondary effects of the dystrophin mutation, such as loss of DGC proteins, complicate the dystrophic pathophysiology. Neuronal nitric oxide synthase (nNOS) is normally localized to the sarcolemma via associations with other DGC proteins [12]. However, in dystrophin-deficient muscle, nNOS is absent from the sarcolemma and greatly downregulated in the cytoplasm [12], [13]. The loss of muscle nNOS has significant physiological effects because its metabolic product, nitric oxide (NO), is a signaling molecule with diverse, systemic functions. Most relevant to the dystrophic muscle pathology, NO is a potent anti-inflammatory molecule that can inhibit the infiltration and activation of inflammatory cells [14], [15] as well as scavenge cytolytic molecules produced by inflammatory cells [16]. Our previous work shows that the loss of nNOS in dystrophic muscle results in significant inflammation, inflammatory cell-mediated damage and fibrosis [17], [18].

Normalizing NO production by expressing a nNOS transgene in dystrophin-deficient *mdx* mouse muscle and cardiac tissue significantly ameliorates the dystrophic pathology. The *mdx* mouse is a naturally-occurring model of DMD that carries a premature stop codon in the dystrophin gene [19] and displays similar disease characteristics to DMD patients including progressive weakness, fiber damage, inflammation, fibrosis and cardiac pathology [17], [20]–[22]. Expression of a nNOS transgene in *mdx* skeletal muscle decreased infiltration of cytotoxic macrophages and reduced subsequent lysis of muscle fibers thereby significantly attenuating the histopathology [17]. Myocardial expression of a nNOS transgene completely prevented cardiac fibrosis in aged *mdx* mice and normalized or improved aspects of the cardiac pathology that are often fatal [18]. These benefits of nNOS transgene expression likely resulted from reductions in profibrotic, inflammatory cells and restoration of competition between nNOS and arginase for their common substrate, arginine [9], [10]. The lack of nNOS activity in dystrophic muscle creates increased arginine availability for metabolism by arginase-expressing, M2 macrophages as well as a loss of metabolites normally generated from oxidation of arginine by nNOS that inhibit arginase activity [9], [23]–[26]. Metabolism of arginine by arginase activates pro-fibrotic pathways and promotes connective tissue deposition in muscle [10], [27]–[30]. When the nNOS transgene is expressed in dystrophic muscle, arginine can be diverted from the pro-fibrotic, arginase pathway and metabolized by nNOS to generate NO for protective functions [10]. These observations show that increasing nNOS expression and activity can ameliorate components of the dystrophin-deficient pathology that contribute to mortality suggesting that the nNOS transgene could affect life-span.

Though the *mdx* dystrophy is genetically homologous to DMD and shares many characteristics of the human disease, DMD and *mdx* pathologies differ distinctly. For example, the DMD is characterized by progressive muscle wasting that results in premature death. However, the *mdx* mouse undergoes significant regeneration following an early phase of necrosis and lives a nearly normal life-span [21], [31] making it impractical for analyses in which survival is an outcome measure. The mild, *mdx* phenotype is attributed to endogenous upregulation of the dystrophin homologue, utrophin, at the sarcolemma where it can functionally compensate for the loss of dystrophin [32]–[33]. Mice that lack expression of both dystrophin and utrophin (double knockout (dko)) experience a very severe disease course that phenotypically resembles the DMD dystrophy despite the genetic heterogeneity of the two pathologies. Dko mice experience an early disease onset characterized by severe and progressive atrophy, weight loss after weaning, contractures, kyphosis, respiratory difficulty, cardiac involvement and early death by 14 weeks of age [20], [34], [35].

In this investigation, we tested whether increasing nNOS expression improves survival of dko mice. Our previous studies using dystrophin-deficient mice showed that reductions of nNOS and NO in dystrophic muscle and heart significantly contribute to multiple elements of the pathology including inflammatory cell-mediated muscle fiber damage, fibrosis and cardiac dysfunction [10], [17], [18]. We hypothesize that NO-mediated amelioration of these components of the pathology will extend the life-span of dko mice. We tested our hypothesis by generating nNOS transgene-expressing mice that were deficient in dystrophin and utrophin (nNOS Tg+/dko) and analyzing the effects of the transgene on survival, fibrosis, inflammation and muscle damage.

## Materials and Methods

### Mice

All experiments involving animals were performed according to the National Institutes of Health Guide for the Care and Use of Laboratory Animals. The protocols were approved by the Animal Research Committee of the University of California, Los Angeles (AWA Assurance #A3196-01). Mice were housed in the UCLA vivarium and examined daily for signs of distress, injury or disease. Mice who displayed signs of sickness or distress such as dehydration, inability to locomote or skin ulceration were euthanized by isoflurane inhalation.

Mdx female mice heterozygous for utrophin (mdx;utr+/−; gift from Dr. Mark Grady (Washington University, St. Louis) and Dr. Joshua Sanes (Harvard University, Cambridge)) were bred with muscle-specific nNOS transgenic males [17] on an mdx background (nNOS Tg+/mdx) to generate F1 offspring that are all dystrophin-deficient with some heterozygous for utrophin and nNOS transgene expression (nNOS Tg+/−;mdx;utr+/−). The F1 male nNOS Tg+/mdx;utr+/− mice were identified by PCR using genomic tail DNA as previously described [17], [36], [37] and back-crossed with mdx;utr+/− females. The resulting F2 litters were screened to identify the dystrophin and utrophin knock-outs (dko) with (nNOS Tg+/dko) and without (nNOS Tg-/dko) nNOS transgenic expression that were subsequently used for experimentation. The nNOS transgenic line used in these studies shows transgene expression in skeletal and cardiac muscle [18].

### nNOS expression

Whole extracts of tibialis anterior muscle were loaded to 10% acrylamide gels (30 µg protein per lane as determined by the method of Minamide [38]) and electrophoresed according to Laemmli [39]. Following electrophoretic transfer to nitrocellulose membrane while immersed in transfer buffer (39 mM glycine, 48 mM Tris, 0.037% sodium dodecyl sulfate, 20% methanol) [40], loading uniformity and transfer efficiency were assessed by staining with 0.1% Ponceau S (Sigma, St. Louis, MO). Subsequently, the membranes were incubated in blocking buffer (0.5% Tween-20, 0.2% gelatin and 3% dry milk) for one hour at room temperature to inhibit non-specific binding. The membranes were probed with mouse anti-nNOS (Transduction Labs, Franklin Lakes, NJ) for 2 hours at room temperature followed by horseradish peroxidase (HRP)-conjugated anti-mouse IgG (Amersham, Piscataway, NJ) for one hour. Membranes were washed with 0.1% Tween in PBS between incubations. nNOS bands were visualized using enhanced chemiluminescence and expression was quantified densitometrically using imaging software (Alpha Innotech, San Leandro, CA).

### nNOS localization

Mice were euthanized at 51–53 days of age via isoflurane inhalation and tissues for histological analysis were collected immediately and frozen in isopentane at −80°C until being cut into 10 µm cross-sections. Sections were air-dried and fixed in cold acetone, followed by incubation with 2% gelatin and 3% bovine serum albumin in phosphate buffered saline (PBS) to prevent non-specific binding of antibodies. Sections were then incubated with rabbit anti-human nNOS (Serotec, Oxford, UK) overnight at 4°C, for 1 hour at room temperature with a biotinylated anti-rabbit secondary antibody (Vector Laboratories, Burlingame, CA) and then for 30 minutes at room temperature with Texas Red-labeled avidin D (Vector Laboratories). Sections were washed with PBS between incubations.

### Nitric oxide detection

Nitric oxide was detected in situ using 4,5-diaminofluorescein diacetate (DAF-2 DA, EMD Chemicals, San Diego, CA) according to Heydemann et al. [41]. Ten micron-thick, frozen, quadriceps sections were incubated with 100 µM DAF-2 DA in DMEM containing 10% fetal bovine serum and 500 µM L-arginine (EMD) for 30 minutes at 37°C. Sections were then washed in PBS twice, for 5 minutes and mounted. Signal specificity was confirmed by treating negative control slides with 1 mM N-nitro-L-arginine methyl ester hydrochloride (Sigma) to inhibit NOS activity. DAF-2 DA staining was visualized and quantified under fluorescent optics. A pre-determined and random pattern containing 5 areas and totaling at least 1000 fibers was quantified in each section. The intensity of fluorescence in each fiber was measured via digital imaging (Bioquant, Nashville, TN) by sampling from an 8 µm diameter circle and was corrected for background levels by measuring the signal from an area of the slide with no tissue and subtracting that background value from the cytosolic fluorescence measurements. Measurements and images were obtained below saturation levels with exposure kept constant for all samples.

### Quantitative immunohistochemistry

Tissues were collected and stored in isopentane as described above and quadriceps, solei, hemi-diaphragms and hearts were cut into 10 µm cross-sections. Sections were then air-dried and fixed in cold acetone, and endogenous peroxidase activity was quenched with 0.03% hydrogen peroxide. Sections were incubated with 2% gelatin and 3% bovine serum albumin in PBS to prevent non-specific binding of antibodies. Primary antibodies were applied for 2 hours at room temperature in a humidified chamber followed by host-appropriate biotinylated secondary antibodies (Vector Laboratories) for 30 minutes at room temperature and finally, HRP-avidin D for 30 minutes. Sections were washed with PBS between antibody incubations. Labeled cells were visualized using 3-amino-9-ethyl carbazole (AEC, red) (Vector Laboratories) as substrate. Immunofluorescent labeling was performed using FITC- or Texas Red-conjugated secondary antibodies (Vector Laboratories) for 30 minutes at room temperature following incubation with the primary antibody as described above. ProLong Gold mounting medium (Invitrogen, Carlsbad, CA) containing DAPI was used to conterstain nuclei. Antibodies used for inflammatory cell-staining were anti-mouse CD4 from supernatants of hybridoma cultures (hybridomas obtained from American Type Culture Collection, Bethesda, MD), rat monoclonal anti-mouse CD8 (Southern Biotech, Birmingham, AL), anti-mouse CD68 (Serotec, Raleigh, NC), polyclonal rabbit anti-mouse eosinophil granule major basic protein (gift from Dr. J.J. Lee, Mayo Clinic Scottsdale, AZ) [42] and anti-mouse CD163 (Santa Cruz, Santa Cruz, CA). Although CD68 is reported to be expressed by all macrophages in humans and in circulating populations in rodents, empirical findings show that CD68 expression in injured rodent muscle is selective for a macrophage subpopulation that invades injured tissue rapidly, is phagocytic and cytotoxic, reflecting M1 activation [43], [44], [45]. CD163 was selected as an M2 macrophage marker because CD163+ macrophages are activated by Th2 cytokines such as IL-10 and IL-4, they express transcripts associated with M2 activation and they invade injured muscle subsequent to invasion by CD68+ M1 macrophages [44], [46], [47].

The concentrations of inflammatory cells were measured by histomorphometry by an investigator who was blinded to the genotype of each sample. The total number of positively stained cells in an entire cross-section from each sample was counted and the area of the cross-section was measured using an eyepiece containing a calibrated 10×10 grid so that the concentration of cells per volume of the tissue section could be calculated using the known thickness (10 µm) of the section, as previously described [48].

### Fibrosis assay

Connective tissue content of quadriceps, solei, hearts and diaphragms was quantified by measuring the amount of hydroxyproline in the tissues according to the technique of Kivirikko et al. [49] that we have used previously [18].

Immunofluorescent labeling for collagen type 1 was performed on 10 µm-thick tissue sections. Sections were fixed and non-specific binding of antibodies was prevented as described above. Sections were incubated with rabbit anti-rat collagen type 1 (Chemicon, Temecula, CA) for three hours at room temperature followed by a Texas Red-conjugated anti-rabbit secondary antibody (Vector) for 30 minutes at room temperature. Sections were washed with PBS between antibody incubations.

### Fiber damage assays

Lesions in soleus muscle fibers were assayed two ways by measuring the presence of the fluorescent, extracellular tracer day, procion orange in the cytosol of the muscle fibers. Procion orange is a vital dye that is not actively transported across cell membranes, instead entering through membrane lesions. Thus, its presence in the cytosol indicates an injured fiber in which there is unregulated transit of large molecules across the membrane. Freshly-dissected soleus muscles were mounted at rest length and incubated in 0.5% procion orange in Kreb's Ringer solution for 1 hour at room temperature and then washed with Kreb's Ringer before being frozen in isopentane and subsequently cut into 10 µm-thick cross-sections. The sections were viewed microscopically using fluorescent optics and the bright fibers indicating the presence of membrane lesions were counted and expressed as a percentage of the total number of fibers in a given section. We also measured the fluorescence intensity of each fiber in a cross-section using a digital imaging system (Bioquant) as described above.

### Statistics

Statistical differences between experimental groups were determined using the two-tailed Mann-Whitney test. Survival data are displayed as Kaplan-Meier plots and statistical significance between the curves was tested using the Log Rank test. Significant differences in survival at a specific age or with regard to mean life-span were tested using the χ^2^ test or the Mann-Whitney U test, respectively. P-values were set at 0.05.

## Results

### nNOS transgene expression increases survival in dko mice

**Figure 1:**
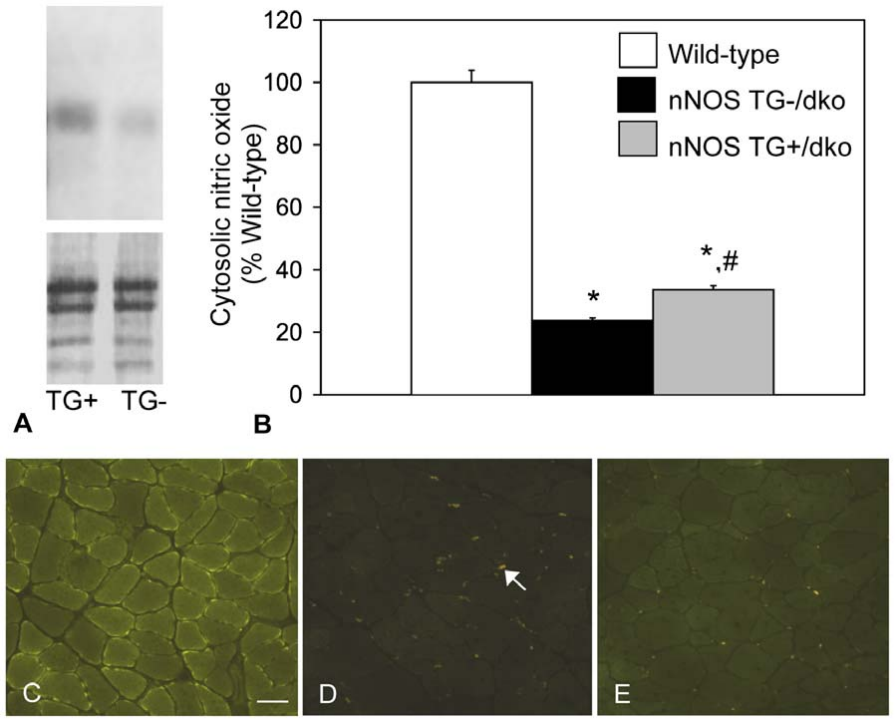
nNOS protein and nitric oxide are increased in dko mice expressing a nNOS transgene. A. Overexpression of the nNOS transgene was confirmed by western blots for all mice assayed in the investigation. Upper panel shows a blot for nNOS in a representative nNOS TG+/dko muscle extract (Tg+) and a representative nNOS TG-/dko extract (TG-). Lower panel shows the same blot stained with Ponceau red prior to antibody labeling to confirm uniform loading of samples. B. Percent cytosolic nitric oxide (NO) in wild-type (white bar), nNOS TG-/dko (black bar) and nNOS TG+/dko (grey bar) muscle. n = 5 mice per group. Error bars represent standard error of the mean. *  =  significant difference as compared to wild-type. #  =  significant difference as compared to nNOS TG-/dko. p<0.05. C. Wild-type muscle showing NO present in the cytosol and highly concentrated at the sarcolemma. Bar = 50 µm. D. nNOS TG-/dko muscle exhibits low levels of NO in muscle fibers, but strong NO production by infiltrating mononuclear cells (arrow). E. Increased levels of NO are evident in the cytosol of muscle fibers from nNOS TG+/dko mice.

**Figure 2:**
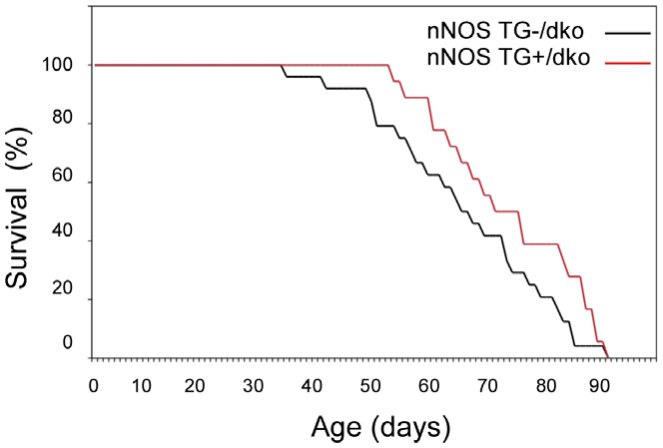
nNOS transgene expression significantly increases survival in dko mice. Survival of nNOS TG-/dko mice (black line, n = 24) and nNOS TG+/dko mice (red line, n = 18) is plotted as Kaplan-Meier curves. The survival curve of nNOS TG+/dko mice is significantly different from nNOS TG-/dko mice at p<0.05.

Life-span data were collected from nNOS TG+/dko and nNOS TG-/dko mice for which transgene expression or absence was confirmed by western blots (Figure 1A.). Densitometric analysis of western blots showed that the nNOS TG+/dko mice exhibit a 200% increase in nNOS expression as compared to nNOS TG-/dko mice (n = 5 per group). Likewise, production of NO was increased by 42% in muscle from dko mice expressing the nNOS transgene (Figure 1B–E). The Kaplan-Meier survival curve of the nNOS TG+/dko mice was significantly different from the nNOS TG-/dko mice showing that the nNOS transgene improved survival (p = 0.048) (Figure 2) and produced a significant, 12% increase in mean life-span (67 days, *sem* = 2.4 vs. 75 days, *sem* = 2.9; p = 0.041). The nNOS transgene was also associated with a delay in the onset of mortality with the first nNOS TG-/dko mouse dying at 35 days of age while the first nNOS TG+/dko mouse did not die until 54 days of age. Based on these findings, we performed all subsequent experimentation on nNOS TG+/dko and nNOS TG-/dko mice at 51–53 days of age because 100% of the nNOS TG+/dko population was alive and survival between the two groups differed significantly at this time point (p = 0.044) indicating that the nNOS transgene affected onset of the fatal pathology.

### Expression of a nNOS transgene does not affect muscle fiber lysis in dko mice

**Figure 3:**
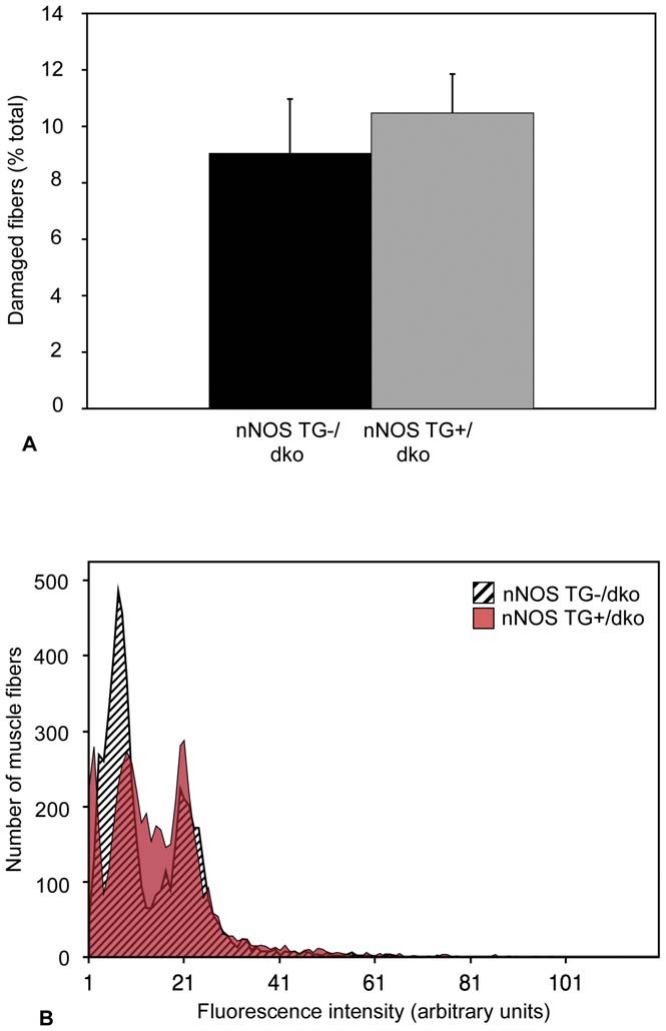
nNOS transgene expression does not reduce muscle fiber damage in dko mice. A. The percentage of damaged fibers in soleus muscles of nNOS TG-/dko mice (black bar) and nNOS TG+/dko mice (grey bar) does not differ. n = 5 mice per group. B. The aggregate distribution of intracellular fluorescence in all soleus fibers from each group. A rightward shift on the abscissa would indicate an increase in the number of fibers with lesions. The black and white, striped peaks represent fibers of nNOS TG-/dko mice, the red peaks represent fibers from nNOS TG+/dko mice and the striped, red areas indicate overlap between the groups. n = 5 mice per group.

Assays of muscle fiber damage indicated by the unregulated influx of extracellular marker dye into the cytosol of muscle fibers showed that nNOS transgene expression had no significant effect on muscle fiber injury in dko mice (Figure 3). Both nNOS TG+/dko and nNOS TG-/dko mice showed a biphasic distribution of fiber injury that resembled the distribution of injury that was previously observed in 12-week-old *mdx* muscle [47], indicating that the biphasic distribution does not require utrophin mutation. The lack of effect of nNOS transgene expression on the magnitude of muscle fiber injury is in contrast to findings in *mdx* mice in which expression of the nNOS transgene resulted in a 70% decrease in muscle fiber lysis due to decreased macrophage infiltration [17].

### nNOS TG+/dko mice show a specific reduction in pro-fibrotic, M2c macrophages that is associated with a significant decrease in muscle fibrosis

**Figure 4:**
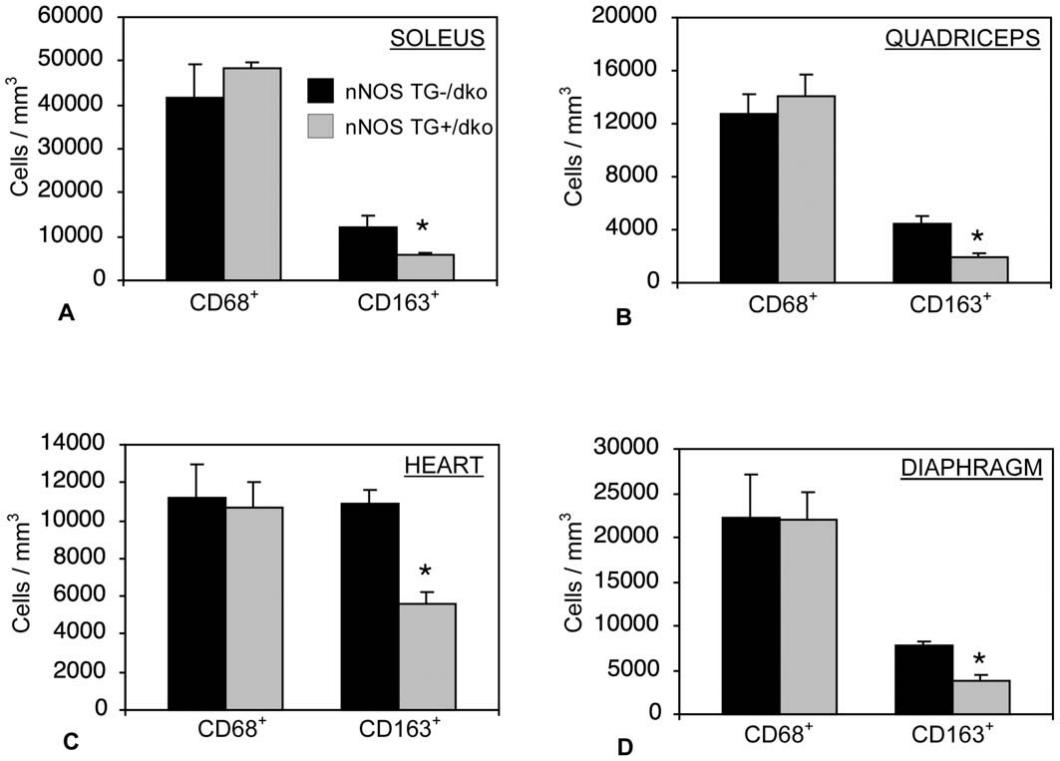
nNOS reduces pro-fibrotic, CD163+ macrophage concentrations in dko skeletal and cardiac muscle. The concentration of CD68+ and CD163+ macrophages was quantified in soleus (A), quadriceps (B), heart (C) and diaphragm (D) muscles of nNOS TG-/dko mice (black bars) and nNOS TG+/dko mice (grey bars). *  =  significant difference in cell concentration as compared to non-transgenic mice at p<0.05. n = 5 mice per group.

**Figure 5:**
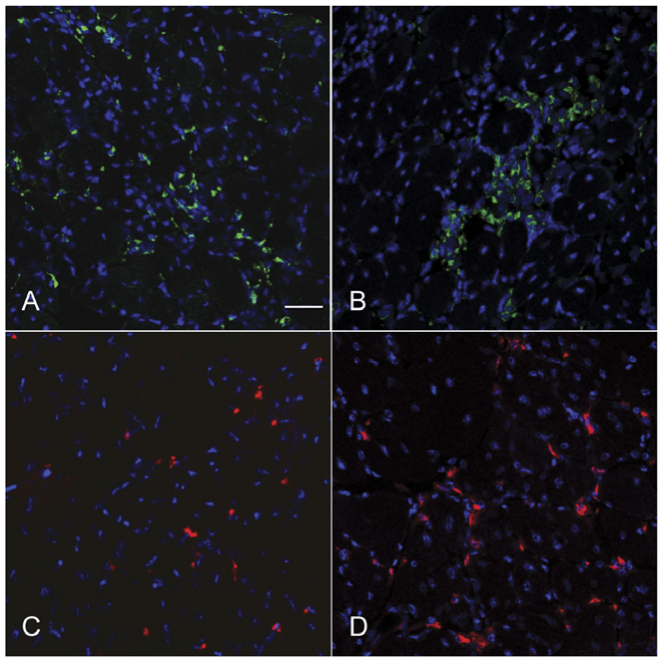
CD163+ macrophage infiltration is decreased in nNOS TG+/dko muscle. CD68+ (green) and CD163+ (red) macrophages were immunolabeled in quadriceps sections. Nuclei were counterstained with DAPI (blue). There is no difference in the number of CD68+ macrophages between nNOS TG+/dko (A) and nNOS TG-/dko (B) muscles. However, there are fewer CD163+ macrophages in nNOS TG+/dko muscle (C) as compared to nNOS TG-/dko muscle (D). Bar = 50 µm.

**Figure 6:**
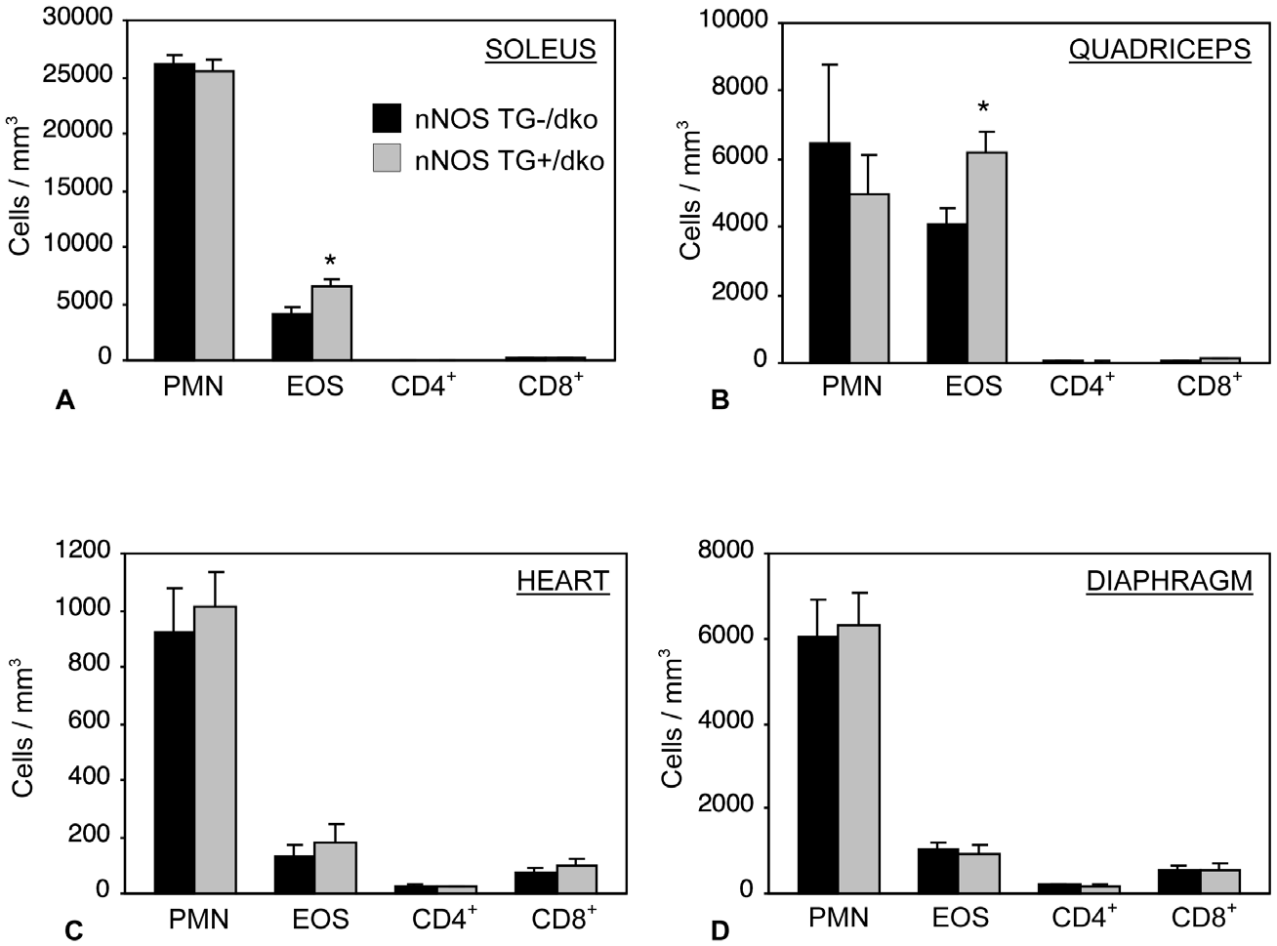
Neutrophil, eosinophil, CD4+ and CD8+ cell concentrations are not decreased in nNOS TG+/dko mice. A. Inflammatory cell counts from soleus muscle. PMN  =  neutrophils, EOS  =  eosinophils, CD4^+^  =  CD4^+^ cells, CD8^+^  =  CD8^+^ cells. Black bars  =  nNOS TG-/dko, grey bars  =  nNOS TG+/dko. B. Inflammatory cell counts from quadriceps muscles. C. Inflammatory cell counts from cardiac muscle. D. Inflammatory cell counts from diaphragm muscle. n = 5 mice per group. *  =  significant difference as compared to same muscle from non-transgenic mice at p<0.05. Some error bars are too small to visualize.

Because nNOS transgene expression did not reduce muscle fiber injury in dko muscle although previous work showed that expression of the transgene in *mdx* muscle reduced fiber damage and the numbers of cytolytic macrophages in muscle, we tested whether the nNOS transgene affected inflammation in dko muscles. We found that the concentration of M1 macrophages, labeled with anti-CD68, was not changed with expression of the nNOS transgene (control vs. nNOS TG+/dko, soleus: 41,859 cells/mm^3^, SEM = 9428 vs. 48,191, SEM = 7272, quads: 12,685 cells/mm^3^, SEM = 1567 vs. 14,017, SEM = 1651), heart (11,228 cells/mm^3^, SEM = 1739 vs. 10,713 cells/mm^3^, SEM  = 1364) and diaphragm: (22,324 cells/mm^3^, SEM = 4696 vs. 21,938 cells/mm^3^, SEM = 3120) (Figures 4 and 5). However, the concentration of CD163+, M2 macrophages was significantly reduced in nNOS TG+/dko muscles (soleus: 12,288 cells/mm^3^, SEM = 2362 vs. 5762, SEM = 418; quads: 4389 cells/mm^3^, SEM = 577 vs. 1860, SEM = 381; heart: 10,918 cells/mm^3^, SEM = 655 vs. 5645 cells/mm^3^, SEM  = 612; diaphragm: 7808 cells/mm^3^, SEM = 431 vs. 3819 cells/mm^3^, SEM = 623) (Figures 4 and 5). We did not observe any change in the concentrations of neutrophils, CD4+ or CD8+ T-cells associated with nNOS transgene expression, although we noted a significant increase in eosinophils in the hindlimb muscles of nNOS TG+/dko mice as compared to controls (soleus: 4161 cells/mm^3^, SEM = 515 vs. 6592 cells/mm^3^, SEM = 521; quadriceps: 4052 cells/mm^3^, SEM = 500 vs. 6175 cells/mm^3^, SEM = 601) (Figure 6).

**Figure 7:**
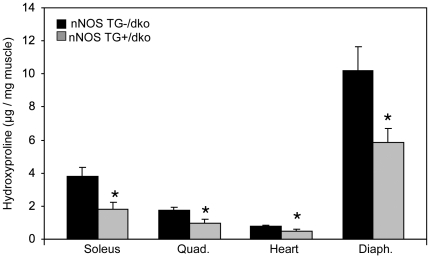
Skeletal and cardiac muscle fibrosis is significantly reduced in dko mice expressing a nNOS transgene. Connective tissue content of soleus, quadriceps (Quad.), heart and diaphragm (Diaph.) muscles from nNOS TG-/dko mice (black bars) and nNOS TG+/dko mice (grey bars) was quantified by measuring hydroxyproline levels. * indicates significant difference from nNOS TG-/dko at p<0.05. n = 5 mice per group.

**Figure 8:**
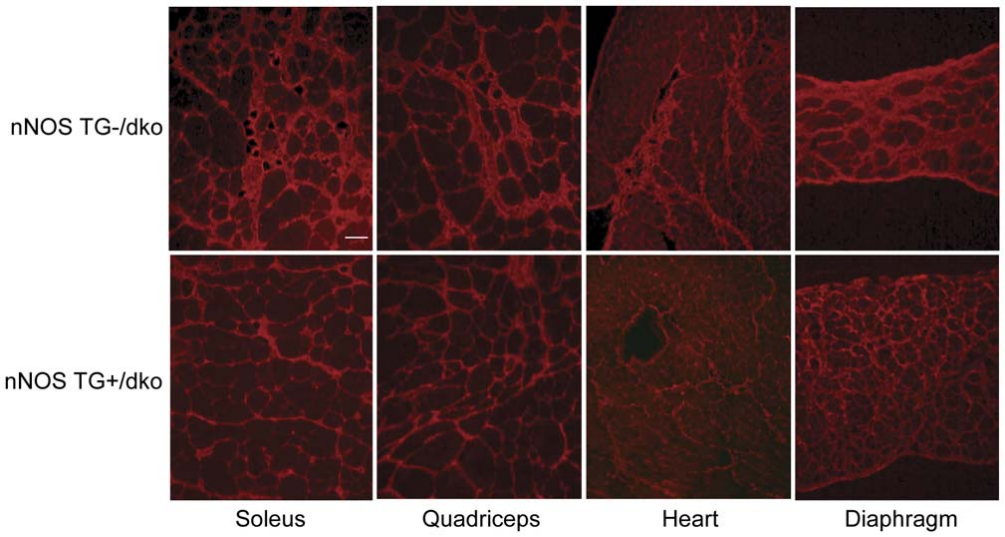
Fibrotic lesions are reduced in skeletal muscle and heart of nNOS TG+/dko mice. Immunofluorescent labeling for collagen type 1 in soleus, quadriceps, heart and diaphragm muscles revealed less fibrosis in dko mice expressing the nNOS transgene. Top row  =  nNOS TG-/dko tissues. Bottom row  =  nNOS TG+/dko tissues. Bar = 50 µm.

**Figure 9:**
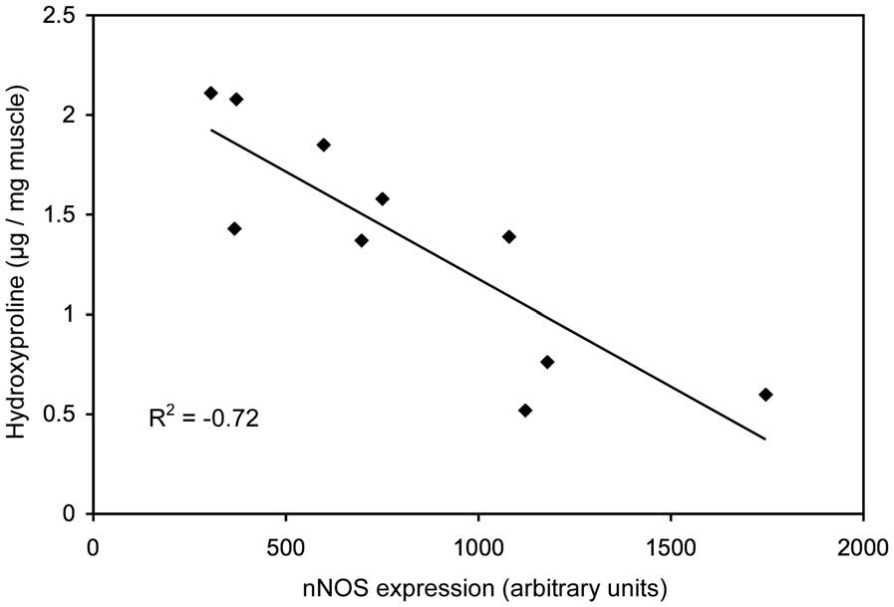
Fibrosis is inversely correlated with nNOS expression in dko muscle. Linear regression was used to quantify the relationship between nNOS expression (measured densitometrically following western blotting) and hydroxyproline content in quadriceps muscles from nNOS TG+/dko and nNOS TG-/dko mice. n = 5 mice per group.

Since M2 macrophages express arginase and promote fibrosis, we assayed whether the reduction of CD163+ macrophages affected muscle fibrosis in dko mice expressing the nNOS transgene. The nNOS TG+/dko mice experienced significantly less fibrosis in soleus, quadriceps, heart and diaphragm muscle suggesting that the nNOS transgene reduced fibrosis by modulating the M2 macrophage numbers (Figures 7 and 8). The relationship between nNOS and dystrophic fibrosis is further illustrated by linear regression analysis showing that nNOS expression is inversely correlated with fibrosis in dko mice (Figure 9). These data suggest that the decrease in fibrosis could contribute to the increased survival rate and lifespan of the nNOS TG+/dko mice.

### nNOS protein is cytosolic in nNOS transgenic, dko mice

**Figure 10:**
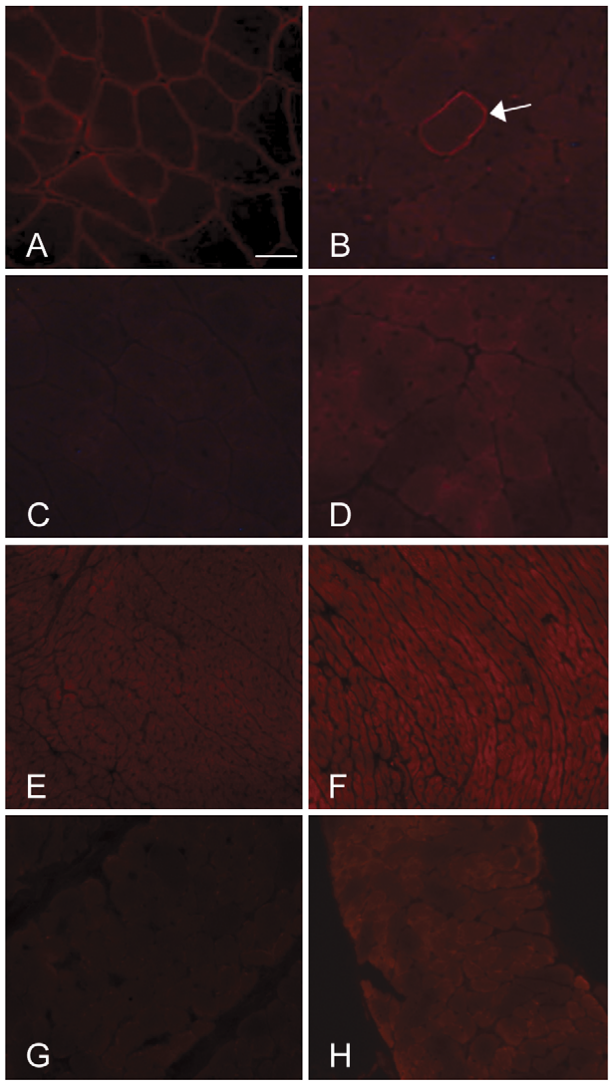
nNOS expression is localized to the cytosol in nNOS TG+/dko mice. Immunofluorescent labeling for nNOS in hindlimb, cardiac and respiratory muscles. A. nNOS is present at the sarcolemma in C57 quadriceps. B. A single, revertant fiber (arrow) in nNOS TG+/dko quadriceps shows sarcolemmal localization of nNOS as opposed to the cytosolic staining throughout the section. C, E, G. nNOS TG-/dko quadriceps (C), heart (E) and diaphragm (G) muscles show no sarcolemmal nNOS expression and barely detectable to low levels of cytosolic nNOS expression. D, F, H. Increased levels of cytosolic nNOS expression are detectable in quadriceps (D), heart (F) and diaphragm (H) muscles of nNOS TG+/dko mice. Bar  =  50 µm.

Most nNOS protein in skeletal muscle is normally localized at the cell membrane as part of the dystrophin protein complex via interactions with syntrophin [50]. However, nNOS is dramatically down-regulated in both the membrane and cytosolic compartments in dystrophin-deficient muscle [12], [13]. Recent publications propose that restoration to the sarcolemma is necessary for functional nNOS signaling in dystrophic muscle [51], [52]. On the contrary, we did not detect nNOS protein at the sarcolemma in the nNOS TG+/dko muscle, but detected nNOS only in the cytosol with increased nNOS expression in nNOS TG+/dko hindlimb, cardiac and respiratory muscles (Figure 10). From this observation we conclude that sarcolemmal localization is not required for nNOS to be beneficial in reducing the dystrophic pathology and most importantly, extending survival in nNOS TG+/dko mice.

### Body mass and muscle mass of dko mice are not affected by nNOS transgene expression

**Figure 11:**
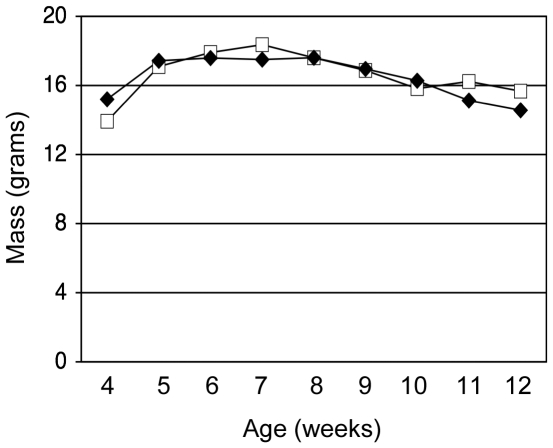
nNOS transgene expression does not prevent atrophy in dko mice. Body masses of nNOS TG-/dko (open squares, n = 9) and nNOS TG+/dko (filled triangles, n = 8) mice are plotted as a function of time and show no difference between groups. There was no significant difference between the body masses of males and females within each group, so data from both genders were combined.

Reflective of severe skeletal muscle disease and atrophy, dko mice are smaller than wild-type [53] and *mdx* mice [34], [35] and experience continuing weight-loss prior to death [35]. Attenuation of body mass loss has been observed in dko mice with prolonged survival due to expression of dystrophin complex proteins, including nNOS [54], which indicates a decrease in muscle atrophy that could be a factor in extending life-span. We determined whether similar benefits occurred in the nNOS TG+/dko mice since nNOS expression was increased and the mice lived longer. However, body mass and muscle mass of the nNOS TG+/dko mice did not differ from non-transgenic controls (Figure 11, Table 1) showing that amelioration of muscle atrophy does not contribute to the nNOS transgene-associated increase in life-span.

**Table 1:** Body and muscle masses of nNOS TG- and nNOS TG+/dko mice.

	Body mass	Quad.	TA	Soleus	Gastroc.	Heart
nNOS TG-/dko	18.6 (0.89)	124.5 (12)	34.9 (1.8)	8.4 (0.67)	87.6 (8.1)	74.0 (7.2)
nNOS TG+/dko	16.2 (0.76)	112.1 (7.5)	36.0 (1.9)	9.1 (0.78)	90.4 (6.2)	86.0 (4.0)

Masses are in grams (standard error of the mean) and were measured at 51–53 days of age. nNOS TG-/dko  = 9 mice. nNOS TG+/dko  = 8 mice. Quad.  =  quadriceps, TA  =  tibialis anterior, Gastroc.  =  gastrocnemius. There were no significant differences between male and female body or muscle masses in a group, so values were combined.

## Discussion

Our findings show that deficits in nNOS expression and NO production contribute to a shortened life-span in a severe model of DMD, the dystrophin/utrophin double knockout mouse. While deficiencies in nNOS are secondary to the disease etiology, lack of nNOS protein and the signaling effects of its metabolic product, NO, can promote multiple components of the pathophysiology including inflammation [17], muscle fiber lysis [17], defects in neuromuscular junction structure [55], fibrosis [18], cardiac functional deficits [18], [20], and misregulation of intramuscular blood flow [56], [57]. By expressing nNOS from a muscle-specific transgene, we were able to significantly delay the onset of the fatal pathology and extend life-span in dko mice. The increased survival of nNOS TG+/dko mice was associated with a significant reduction in pro-fibrotic, M2c macrophages and connective tissue content of skeletal muscle and cardiac tissue suggesting that the nNOS transgene enhanced longevity by attenuating fibrosis.

Surprisingly, expression of the nNOS transgene had different effects in dko and *mdx* mice. Our previous work showed that nNOS transgene expression in muscle of *mdx* mice significantly reduced muscle fiber damage [17]. This decrease in muscle lysis was attributed to a nNOS transgene-induced reduction in cytotoxic muscle macrophages. Since total macrophage concentrations in nNOS TG+/dko were reduced by magnitudes comparable to those in nNOS TG+/mdx mice, we anticipated a similar effect on fiber lysis. However, muscle fiber damage was not attenuated in the nNOS TG+/dko mice indicating that a reduction of muscle necrosis did not contribute to the increase in dko lifespan. Instead, this suggested a functional distinction between the macrophage populations present in *mdx* and dko muscles. Accordingly, we confirmed that the nNOS transgene did not affect the concentration of cytolytic, CD68+ M1 macrophages in dko mice but had a significant and specific effect in reducing the concentration of pro-fibrotic, CD163+ M2c macrophages. The different effects of the nNOS transgene are likely due to differences in the disease processes between dko and *mdx* mice at the times sampled. The 4-week time point in *mdx* mice is dominated by pro-inflammatory signals and represents the acute peak of pathology with significant muscle necrosis, whereas the time point we sampled in dko mice is largely pro-fibrotic and indicative of the disease's end-stage. The difference in magnitude of nNOS overexpression between the mdx and dko strains could also contribute to the variable effects on the pathologies. The dko mice expressing the nNOS transgene experience a 200% increase in nNOS expression whereas mdx mice expressing the same transgene demonstrate a 500% increase in nNOS expression (data not shown). While significant benefits were observed in the nNOS TG+/dko mice, nNOS expression was only 17% of that in wild-type mice. Also unexpected, nNOS TG+/dko hindlimb muscles showed higher eosinophil concentrations than nNOS TG-/dko hindlimb muscle, despite previous findings that eosinophils can promote fibrosis in *mdx* muscle [58]. However, increased NO generated by the transgenic nNOS protein may have stimulated eosinophil migration, as previously demonstrated *in vitro* using human eosinophils [59].

Increased fibrosis that is associated with nNOS deficiency relates closely to the early death of dko mice. Pathological deposition of connective tissue is an underlying factor in the two primary causes of death in DMD: respiratory and cardiac dysfunction [60]–[63]. The dko mouse similarly experiences these aspects of the dystrophic pathology validating its use as a model for the disease. Previous studies showed that dko mice exhibit increased cardiac fibrosis, stiffness and conduction defects that replicate those observed in DMD patients [20], [35], [64], [65]. Likewise, dko mice develop severe fibrosis of the diaphragm [35] causing respiratory impairment that is characteristic of the dystrophic pathology [66]. Since both clinical and experimental findings indicate that fibrosis is a primary factor in the respiratory dysfunction and cardiomyopathy that are the leading causes of death in dystrophin-deficient dystrophy, the extended lifespan of nNOS Tg+/dko mice may reflect the decreased fibrosis that occurred in respiratory and cardiac muscles. We are not able to determine whether the reductions of fibrosis in cardiac, diaphragm or limb muscles all contribute to increasing dko survival, although it has been shown that specific restoration of diaphragm or skeletal muscle function can rescue cardiac function in dko mice [67], [68].

Sarcolemmal localization of nNOS was not required to increase survival of nNOS TG+/dko mice or to decrease cardiac or skeletal muscle fibrosis in the disease. Nevertheless, several previous reports have suggested that nNOS localization to the cell membrane may be a significant feature in reversing other aspects of the dystrophic pathology. For example, mice that are null mutants for α-syntrophin showed a large reduction in sarcolemma-associated nNOS that is accompanied by defects in autonomic regulation of vasoconstriction [69]. However, total nNOS is also reduced in the muscles of α-syntrophin null mice, so whether the defects were attributable to a loss of membrane-associated nNOS *per se* or caused by a loss of total NO production by muscle remains unknown. Similarly, excised muscles from nNOS null mice, that lack both membrane-associated and cytosolic nNOS, showed increased fatigability and reduced force production, which was consistent with the possibility that loss of membrane-associated nNOS caused the defects [52]. However, whether those functional deficits could be reversed by restoring cytosolic nNOS has not been tested. In other studies, slower recovery from exercise and loss of normal regulation of vasodilation were reported in mutant mice that express ε-sarcoglycan instead ofα-sarcoglycan at the muscle membrane [51]. These mice experience loss of nNOS from the sarcolemma and small reductions in total nNOS [51] which could indicate that the specific loss of membrane-localized nNOS rather than reductions in total nNOS were most important in the functional deficits that were reported. However, when the mice were treated with phosphodiesterase 5A (PDE5A) inhibitor, post-exercise fatigue was reduced [51]. Similarly, PDE5A treatments of *mdx* mice reduced post-exercise fatigue and improved vasodilation [51]. Those findings showed that NOS is necessary for normal recovery following exercise because PDE5A inhibits the degradation of cGMP which is a signaling molecule generated downstream of NOS activation; thus, PDE5A treatments can compensate for deficiencies in NO production. However, since neither mouse strain expressed membrane-associated nNOS, the NO-mediated improvement in function in PDE5A-treated mice occurred independently from nNOS localization to the sarcolemma, indicating that increased activation of cytosolic nNOS reduced the functional defects. Furthermore, increased expression of cytosolic nNOS in *mdx* muscle produced great reductions in fatigability during treadmill running [70], showing that increasing total NO production in muscle is sufficient to reduce fatigue, independent of nNOS localization within the cell.

The extent of pathology in dystrophic mice expressing modified dystrophin proteins is also not correlated with sarcolemmal localization of nNOS. Previous studies have shown remarkable success in preventing or reducing disease in dystrophin-deficient mice expressing mini- and micro-dystrophin constructs that induce reorganization of the dystrophin glycoprotein complex [53], [54], [71], [72]. Dystrophin-deficient mice expressing the most effective micro-dystrophin construct, ΔR4-R23, had morphologically normal muscles that were free of fibrosis, necrosis and inflammation and demonstrated normal resistance to contraction-induced injury [71], [72]. Significant levels of nNOS were found in cytosolic fractions of muscles from ΔR4-R23 mice, although nNOS in ΔR4-R23 mdx mice was primarily cytosolic and was only barely detectable in microsomal preparations and minimally expressed at the sarcolemma in tissue sections [72]. Expression of a similar dystrophin construct, ΔR4-R23/ΔC, in utrophin/dystrophin double-mutant mice yielded benefits comparable to those observed with ΔR4-R23 but also failed to restore nNOS to the sarcolemma [53]. Furthermore, the ΔH2-R19, mini-dystrophin construct also generated *mdx* mice with normal muscle morphology and specific force levels [71] while little nNOS was localized to the sarcolemma [72]. In contrast, transgenic *mdx* mice with skeletal muscle expression of Dp260, the retinal isoform of dystrophin, showed significant nNOS expression in muscle membrane fractions yet still exhibited fibrosis, inflammation, degeneration and reduced force production although the pathology was less severe than in *mdx* mice not expressing Dp260 [73]. Interestingly, lack of nNOS at the sarcolemma does not limit voluntary activity in *mdx* mice. The ΔR4-R23 and ΔH2-R19 *mdx* mice were able to run significantly further than wild-type *mdx* mice and as far or further than C57Bl/10, control mice [71]. The dystrophic models described above show that disease phenotype is not correlated with the extent of sarcolemmal nNOS localization, but can be influenced by the characteristics of the dystrophin proteins expressed and the mechanical integrity of the transmembrane complex formed.

Although published findings do not permit conclusions to be made concerning whether nNOS-mediated rescue of dystrophin-deficient muscle is more efficient if nNOS is membrane associated, numerous findings have shown broad, significant, beneficial effects of increased expression of cytosolic nNOS for reducing the pathology of muscular dystrophy. For example, expression of a nNOS transgene in *mdx* muscles that produces an elevation of cytosolic nNOS reduces muscle fiber damage [17], reduces muscle inflammation [17], decreases skeletal muscle and cardiac fibrosis [10], [18], reduces cardiac dysfunction [18], reduces kyphoscoliosis [10], improves neurogenesis [74], provides positive allosteric regulation of phosphofructokinase [70], raises systemic levels of circulating NO [74] and reduces muscle fatigability [70]. Furthermore, elevated NO production caused by increased expression of cytosolic nNOS plays significant regulatory roles in non-dystrophic muscles. For example, increased NO production by cytosolic nNOS modulates the redox balance in muscle and the expression of superoxide dismutase and peroxiredoxin [75] and reduces muscle inflammation and injury during modified muscle use [76]. Notably, all of these beneficial effects were mediated by a transgene that expressed a protein that did not include the µ-peptide that is present in native nNOS in skeletal muscle, showing that nNOS rescue of muscle function does not require expression of the nNOSµ isoform. However, it is feasible but untested that the rescue by increased expression of the nNOSµ isoform may be more, or less, efficient than rescue by the nNOS isoform lacking the µ-peptide.

The current study shows that muscle-specific expression of a nNOS transgene in dko mice has significant benefits that delay the onset of fatal pathology and extend life-span. We determined that nNOS transgene expression significantly decreased fibrosis in cardiac, diaphragm and hindlimb muscles by reducing the concentration of pro-fibrotic macrophages in those tissues. The abatement of fibrosis is likely the primary factor for extending lifespan by improving cardiac and diaphragmatic function. Importantly, the sarcolemmal localization of nNOS was not required to produce these benefits. These findings show that increased nNOS expression in the absence of nNOS targeting to the sarcolemma can significantly ameliorate the pathology and produce clinically relevant outcomes. This knowledge may be therapeutically important because we anticipate that pharmacological or genetic interventions that are directed to increasing total NO production by muscle can be developed much more rapidly than interventions that require targeting nNOS specifically to the cell membrane. We hope that continuing research into the roles of nNOS and NO in dystrophic disease mechanisms will lead to a greater understanding of pathological mechanisms that can ultimately be exploited therapeutically.
